# Phase II study of irinotecan with bolus and high dose infusional 5-FU and folinic acid (modified de Gramont) for first or second line treatment of advanced or metastatic colorectal cancer

**DOI:** 10.1038/sj.bjc.6600641

**Published:** 2002-11-12

**Authors:** P Leonard, M T Seymour, R James, D Hochhauser, J A Ledermann

**Affiliations:** Royal Free and University College Medical School, UCL, London, UK; ICRF Clinical Centre, Cookridge Hospital, Leeds, UK; Christie Hospital, Manchester, UK

**Keywords:** colorectal cancer, irinotecan, 5-fluorouracil (5-FU), irinotecan and 5-FU combination therapy

## Abstract

We investigated the activity of irinotecan given with a more convenient modified bimonthly de Gramont regimen of bolus and infusional 5-fluorouracil [IrMdG] in advanced or metastatic colorectal cancer in the first and second line setting. Irinotecan 180 mg m^−2^ was infused over 90 min. L-folinic acid 175 mg or d,l folinic acid 350 mg was given over 2 h followed by a bolus of 5-fluorouracil (400 mg m^−2^) and a 46 h continuous infusion of 5-fluorouracil (2.4–2.8 g m^−2^). Forty-six previously untreated patients (Group A) and 36 who had received 5-fluorouracil for metastatic disease (Group B) were recruited. Seventy-eight patients were evaluable for response. A partial response was seen in 13 out of 43 (30% [95%CI 28.1–31.9%]) in Group A and 8/35 (23% [95% CI 17.9–28.1%]) in Group B. 40% (95%CI 38.1–41.9%) of Group A and 26% (95% CI 20.9–31.1%) of Group B patients achieved disease stabilisation. The median progression free survival from the start of this treatment was 7 months (95% CI 4.4–9.6 months) in Group A and 5 months (95% CI 2.8–7.2 months) in Group B. Median overall survival was 14 months (95% CI 9.0–18.9) in Group A and 11 months (95% CI 5.9–16.1) in Group B. Grade 3–4 toxicity in both treatment groups were similar; leucopenia 17% and diarrhoea 7–8%. Grade 3–4 mucositis was not seen and severe alopecia affected only three patients. IrMdG is an active and well-tolerated regimen for both the first and second line treatment of advanced colorectal cancer.

*British Journal of Cancer* (2002) **87**, 1216–1220. doi:10.1038/sj.bjc.6600641 www.bjcancer.com

© 2002 Cancer Research UK

## 

Colorectal cancer is the second most common cause of cancer-related death in Europe. For more than 40 years 5-fluorouracil (5-FU) has been the drug most commonly used to treat patients with this disease. The response to this drug varies depending on whether bolus or infusional chemotherapy is given. (Erlichman *et al*, 1988; Advanced Colorectal Cancer Meta-Analysis Project, (1992)) During the last decade it has become clear that biomodulation with folinic acid (FA) and alteration of the scheduling of 5-FU can significantly improve the response rate and toxicity profile (de Gramont *et al*, 1997). Nevertheless, only about 20–30% of patients respond to 5-FU based therapy (Skibber *et al*, 2001) and patients with metastatic disease are not cured by chemotherapy. The topoisomerase I inhibitor, irinotecan, usually given at a dose of 350 mg m^−2^ every 3 weeks or 125 mg m^−2^ weekly for 4 out of 6 weeks, has shown activity in patients with advanced colorectal cancer previously treated with 5-FU. (Rothenberg *et al*, 1996; Rougier *et al*, 1998) Delayed onset diarrhoea, nausea and vomiting, neutropenia, fatigue and alopecia are the main toxicities. Some patients experience an acute cholinergic syndrome. There are now reports that have examined the combination of 5-FU and irinotecan. The combination of two drugs with different modes of action has increased the response rate in advanced colorectal cancer (Douillard *et al*, 2000; Saltz *et al*, 2000). However it is still not clear whether combinations of irinotecan and 5-FU are best used in the first or second line setting.

A ‘Modified de Gramont’ (MdG) regimen has been developed in which a 2-h infusion of a fixed dose of folinic acid is followed by 5-FU bolus then a high dose 46-h 5-FU infusion (de Gramont *et al*, 1998; André *et al*, 1999). This regimen, alone or with oxaliplatin, has now been optimised (Maindrault-Goebel *et al*, 1999; Cheeseman *et al*, 2002). MdG contains a higher dose of 5-FU than the classical de Gramont schedule and avoids the need for hospital attendance on 2 consecutive days. In this pilot study we have investigated a 2 weekly combination of irinotecan given at 180 mg m^−2^ together with MdG in the first and second line setting. The new regimen has been designated IrMdG.

The purpose of this non-randomised study was to investigate the safety and efficacy of IrMdG. It was designed as a pilot study for MRC CR08 (FOCUS), a large randomised trial, now underway, in which two of the experimental arms compare IrMdG in either the first or second line setting.

## METHODS

### Patients

From December 1998 to December 1999, following Institutional Ethical Committee approval and with written informed consent, a total of 82 patients were enrolled. Eligibility criteria were: locally advanced or metastatic colorectal cancer; age 18–75 years; WHO status of 1 or less with a life expectancy of more than 3 months; satisfactory renal function (creatinine 135⩽μmol l^−1^); satisfactory liver function (bilirubin⩽1.5×upper limit of normal (ULN), transaminase⩽5×ULN); adequate haematological function (haemoglobin⩾10 g dl^−1^, absolute neutrophil count ⩾2.0×10^9^ l^−1^ and platelets ⩾150×10^9^ l^−1^). In group A, (*n*=46) patients were required to have measurable or evaluable disease and not to have received any previous chemotherapy for metastatic disease. Patients who had received adjuvant bolus 5-FU/folinic acid were eligible provided chemotherapy had been completed more than 6 months before entry into the study. In group B (*n*=36), the same eligibility criteria applied except that patients had only received 5-FU chemotherapy for metastatic disease, and 4 weeks had to have elapsed since receiving their last dose of 5-FU chemotherapy before entry into the study.

### Statistical analysis

The primary endpoints were the safety and toxicity of this combination regimen. We were able to measure the outcome parameters of response rate, progression-free and overall survival. Progression free survival was defined as the time from the start of treatment until progression or death from disease, or unknown causes. The analysis for toxicity assessment included all 82 patients. The analysis for response criteria included the 78 evaluable patients. This was a non-randomised study so no comparative statistics could be applied. The survival curves are represented as Kaplan-Meier Graphs.

## STUDY DESIGN AND TREATMENT

All patients received irinotecan 180 mg m^−2^ over 90 min by infusion followed by l-folinic acid 175 mg or d,l folinic acid 350 mg intravenously over 2 h. A 5-min bolus dose of 5-FU 400 mg m^−2^ bolus was then given, followed by a continuous infusion of 5-FU 2400–2800 mg m^−2^ over 46 h. The higher dose of 5-FU was given to the first four patients. Following two early deaths the starting dose of 5-FU was reduced to 2400 mg m^−2^. All treatments were given through a central venous line and repeated every 2 weeks. Response assessment took place after six cycles with clinical examination, biochemical evaluation including CEA (carcinoembryonic antigen) measurement and radiological evaluation by computerised tomography or magnetic resonance imaging. WHO response criteria were used. Patients with radiologically confirmed stable or responding disease were offered a further six cycles of treatment at 2 weekly intervals to commence as soon as convenient. Patients were withdrawn from the study if there was excessive toxicity, evidence of progressive disease or at their request. Doses of both drugs were lowered, at the discretion of the treating clinician if excessive toxicity was experienced by the patient. At the end of the treatment period patients were followed up according to local practice and any note of tumour progression was made.

## RESULTS

### Patients and treatments

The characteristics of the 82 patients who met the eligibility criteria for the respective groups are shown in Table 1

**Table 1:**
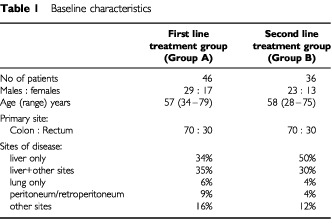
Baseline characteristics

. Figure 1

**Figure 1:**
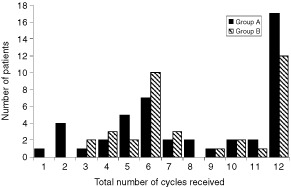
Distribution of treatment cycles received by all patients in the study; first-line treatment (Group A) and second line treatment (Group B).

shows the distribution of all treatment cycles received by the patients on both groups of the study. The number of patients who received at least six cycles of treatment was similar for both Groups of the study, 72% (33 out of 46) for Group A and 81% (29 out of 36) for Group B. Progressive disease was the most common reason for not completing at least six cycles. Three patients experienced severe toxicity after only one cycle. The median number of treatment cycles administered was 8 (range 1–12) for Group A and 7 (range 3–12) for Group B. Fifty-one per cent of those patients in Group A and 41% of Group B patients who completed six cycles went on to complete all 12 cycles.

### Safety

Soon after the initiation of this study, two treatment-related deaths occurred among four patients treated at the higher 5FU dose (2800 mg m^−2^ 46-h infusion). Neither death was clearly dose-related: one patient developed diarrhoea and neutropenia but remained at home without alerting professionals for several days before being admitted moribund; the other was hospitalised with neutropenia, which was beginning to recover when he died of an unexplained cardiac event. Following these events the 5-FU dose was reduced to 2400 mg m^−2^ 46-h infusion for all subsequent patients. The incidence of main toxic effects according to the NCI-CTC grade scale are shown in Tables 2

**Table 2:**
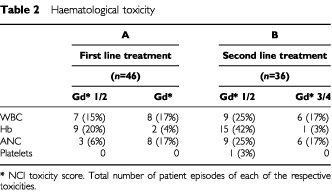
Haematological toxicity

and 3

**Table 3:**
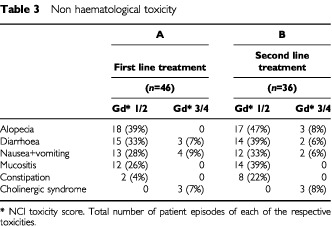
Non haematological toxicity

. Leucopenia was the most common haematological toxicity. The rates of neutropenia in both settings were similar. Four of the eight patients in Group A with Grade 3–4 neutropenia required hospital admission for neutropenic sepsis. This included the two toxic deaths at the start of the study. Only one of seven affected patients in Group B required hospital admission. Non-haematological toxicity was not severe in the majority of cases. No grade 3–4 alopecia was seen in the first line treatment group and only three patients were affected in the second line treatment group. The incidence of grade 3–4 diarrhoea, or cholinergic syndrome requiring atropine, was low compared with published toxicity data from previous studies of single agent irinotecan (Douillard *et al*, 2000). One patient in Group B who experienced grade 3–4 diarrhoea required hospital admission. One other patient in Group B required admission for rehydration following grade 3–4 nausea and vomiting.

## DOSE REDUCTIONS

Seven cycles of the higher dose of 5-FU (2800 mg m^−2^) were given before the dose for all patients was reduced to 2400 mg m^−2^. Overall, 69% (32 out of 46) of Group A patients did not require a dose reduction, and reductions of 15–25% in both cytotoxic drugs were sufficient to keep toxicity within acceptable limits for the remainder. Surprisingly, a greater proportion (83%) of Group B patients was able to tolerate the regimen as second line treatment without a dose reduction. However for the six requiring dose reductions, two patients required a further dose reduction of both drugs. For these patients, reducing the dose of both drugs to 65% of full dose was sufficient to allow treatment to continue.

## RESPONSE RATE AND SURVIVAL

In total, 43 of the 46 patients in Group A were evaluable for response. In addition to the two toxic deaths, there was one other early death from a pulmonary embolism. One patient from Group B developed a bowel fistula and had to be withdrawn from the study. A 30% partial response rate was seen in those patients who had not previously been exposed to 5-FU. A response rate of 23% occurred in those who received this regimen as second line treatment. A larger proportion of patients treated in the first line setting achieved disease stabilisation: 40 *vs* 26% (Table 4

**Table 4:**
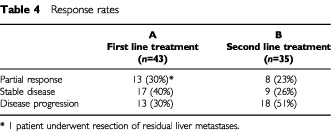
Response rates

).

**Figure 2:**
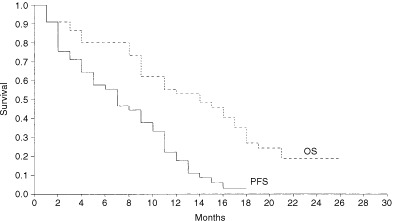
Progression-free survival (PFS) and overall survival (OS) first-line treatment group (Group A). Median PFS is 7 months (95% CI 4.3–9.6 months) and OS is 14 months (95% CI 9.0–18.9 months).

**Figure 3:**
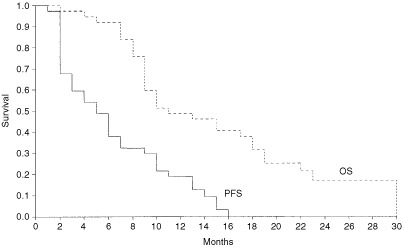
Progression-free survival and overall survival second-line treatment group (Group B). The median PFS is 5 months (95% CI 2.8–7.2 months) and OS is 11 months (95% CI 5.9–16.1 months).

Progression free survival was taken from the start of treatment to the date of disease progression. Median progression-free survival shown in Figure 2

was estimated to be 7 months (95% CI 4.4–9.6 months) in Group A and 5 months (95% CI 2.8–7.2 months) in Group B. Overall survival (Figure 3

) was taken from the date treatment began to the date of death. To date, 15 patients are still alive. Estimated median overall survival for Group A is 14 months (95% CI 9.0–18.9) and for Group B is 11 months (95% CI 5.9–16.1 months).

## DISCUSSION

Bimonthly infusional LV/5-FU (de Gramont regimen) has become a widely accepted treatment with a higher response rate and lower toxicity than bolus LV/5-FU (de Gramont *et al*, 1997). Modification of the de Gramont regimen with folinic acid and bolus 5-FU given only on the first day followed by a 46 h infusion of 5-FU has potential advantages. Patients need to attend hospital only on the first day. This is more convenient for patients and it reduces the hospital and drug costs. There is no consensus about the dose of folinic acid and no evidence for a dose effect in relation to body surface area for this regimen (Ychou *et al*, 1998). We therefore used a fixed dose consisting of a single vial on the first day of each cycle. Several modifications of the de Gramont regimen have been described, and they appear to be as active as the classical regimen (Seymour *et al*, 1998; de Gramont *et al*, 1998) although there has been no randomised comparison.

Preclinical studies suggest that the combination of irinotecan with 5-FU is synergistic. (Guichard *et al*, 1998) Combination studies with the de Gramont regimen have established a 2 weekly dose of irinotecan 180 mg m^−2^ is acceptable (Ducreux *et al*, 1999; André *et al*, 1999). Our study was designed to examine the activity and toxicity of combining irinotecan and the modified de Gramont regimen as first- or second-line therapy of metastatic colorectal cancer. The purpose was to define doses that could be tested in randomised trials comparing combinations of irinotecan and 5-FU, and oxaliplatin and 5-FU. Since the start of the study it has become clear that irinotecan and 5-FU (de Gramont regimen) is active in the first-line therapy of colorectal cancer. A response rate of 34.8% on an intention to treat basis and 3.3 month prolongation in median survival was seen when irinotecan was added to the 5-FU regimen (Douillard *et al*, 2000). The response rate to IrMdG in our study was 30%, which was lower, but the median progression-free survival was 7 months compared to 6.7 months in the randomised trial (Douillard *et al*, 2000). There was very little grade 3/4 non-haematological toxicity. Only 6–7% of patients experienced grade 3/4 diarrhoea and three patients (8%) experienced grade 3/4 alopecia. This was much less than the reported incidence of diarrhoea in the study by Douillard *et al* (2000) However, in their trial, patients received either the classical de Gramont regimen or a weekly high dose infusional 5-FU regimen (Köhne *et al*, 1998). Grade 3/4 neutropenia was 17% with the modified de Gramont regimen, a little lower than that reported by Douillard *et al* (2000), and similar to the level reported by André *et al* (1999). The dose of 5-FU was initially set at 2.8 g m^−2^ over 46 h as the FOLFIRI regimen reported by André *et al* (1999) had used higher doses of 5-FU 2.4–3.0 g m^−2^ in combination with irinotecan. The first four patients enrolled in our study received the higher dose of 5-FU and two died on treatment. The first developed neutropenia and diarrhoea, which was not immediately reported. The second died suddenly from a presumed cardiac arrhythmia while recovering from neutropenia. We therefore reduced the dose of 5-FU to 2.4 g m^−2^ and did not encounter any further life- threatening haematological toxicity.

The second part of the study examined the combination in second-line therapy. The expectation was that the regimen would be less active and more toxic. This was not the case. The response rate was lower at 23% but the median progression-free survival measured from the start of treatment was similar (5 months). Furthermore, 24 (68%) of the 36 enrolled patients had received de Gramont style 5-FU as their previous regimen. In the UK at this time the de Gramont regimen, or a continuous infusion of 5-FU was considered to be the optimal first line regimen for metastatic colorectal (Maughan *et al*, 2002). Other drugs, such as oxaliplatin were unavailable. The pattern of haematological and non-haematological toxicity was similar whether irinotecan and the modified de Gramont regimen were given as first or second-line treatments. The emerging evidence from randomised studies has suggested that combinations of irinotecan and 5-FU should be used in the first-line treatment of colorectal cancer (Douillard *et al*, 2000; Saltz *et al*, 2000). However, the prolongation in median survival from the addition of irinotecan is small. The response rate and similar progression-free survival in the second line setting still raises the question of the optimal use of combinations of irinotecan and 5-FU. The patient cohort that received this regimen in the second line setting had been pretreated with the optimal combination of 5-FU and folinic acid and their median event-free survival was still 5 months. During this time oxaliplatin was not available to patients in the UK who progressed on irinotecan. The Medical Research Council Trial (CR08) (FOCUS- 5-FU, oxaliplatin, CPT-11, use and sequencing) has been designed to address the question of the optimum timing of combination therapies. Bolus 5-FU regimens with irinotecan have been found to cause unexpected early deaths in several National Cancer Institute sponsored co-operative studies. An analysis of deaths in these trials has shown a three-fold increase compared to non-irinotecan regimens (Rothenburg *et al*, 2001). In our experience of IrMdG the toxicity is low (Ledermann *et al*, 2001). The combination of drugs is active in both the first and second line setting and the regimen is now being used as part of the MRC FOCUS trial.
